# CAM-Xenograft Model Provides Preclinical Evidence for the Applicability of [^68^Ga]Ga-Pentixafor in CRC Imaging

**DOI:** 10.3390/cancers14225549

**Published:** 2022-11-11

**Authors:** Katarína Benčurová, Joachim Friske, Maximilian Anderla, Manuela Mayrhofer, Thomas Wanek, Lukas Nics, Gerda Egger, Thomas H. Helbich, Marcus Hacker, Alexander Haug, Markus Mitterhauser, Theresa Balber

**Affiliations:** 1 Ludwig Boltzmann Institute Applied Diagnostics, 1090 Vienna, Austria; 2 Division of Nuclear Medicine, Department of Biomedical Imaging and Image-Guided Therapy, Medical University of Vienna, 1090 Vienna, Austria; 3 Division of Molecular and Structural Preclinical Imaging, Department of Biomedical Imaging and Image-Guided Therapy, Medical University of Vienna, 1090 Vienna, Austria; 4 Department for Inorganic Chemistry, Faculty of Chemistry, University of Vienna, 1090 Vienna, Austria; 5 School of Medical Engineering and Applied Social Sciences, University of Applied Sciences Upper Austria, 4020 Linz, Austria; 6 QIMP Team, Center for Medical Physics and Biomedical Engineering, Medical University of Vienna, 1090 Vienna, Austria; 7 Department of Pathology, Medical University of Vienna, 1090 Vienna, Austria; 8 Comprehensive Cancer Center, Medical University of Vienna, 1090 Vienna, Austria; 9 Christian Doppler Laboratory Applied Metabolomics, 1090 Vienna, Austria

**Keywords:** colorectal cancer, CXCR4, [^68^Ga]Ga-Pentixafor, 2-[^18^F]FDG, in ovo, CAM-xenograft, PET/MRI, HT29, HCT116, CRC imaging

## Abstract

**Simple Summary:**

The high mortality rate of colorectal cancer (CRC) is associated with metastasis to the liver, which is related to an increased expression of the C-X-C chemokine receptor 4 (CXCR4). We hereby report the first preclinical evaluation of [^68^Ga]Ga-Pentixafor, a radiotracer specifically targeting human CXCR4, for CRC imaging. We established the chorioallantoic membrane (CAM)-xenograft model for two different human colon cancer cell lines (HT29 and HCT116) in our facility and conducted a thorough histological characterisation of the obtained xenograft tissues. The subsequently performed simultaneous positron emission tomography and magnetic resonance (µPET/MR) scans demonstrated [^68^Ga]Ga-Pentixafor uptake in CAM-xenografts and provided novel insights into the radiotracer distribution in the chick embryonal organism.

**Abstract:**

Colorectal cancer is one of the leading causes of cancer-related deaths worldwide. Increased expression of CXCR4 has been associated with liver metastasis, disease progression, and shortened survival. Using in vitro cell binding studies and the in ovo model, we aimed to investigate the potential of [^68^Ga]Ga-Pentixafor, a radiotracer specifically targeting human CXCR4, for CRC imaging. Specific membrane binding and internalisation of [^68^Ga]Ga-Pentixafor was shown for HT29 cells, but not for HCT116 cells. Accordingly, [^68^Ga]Ga-Pentixafor accumulated specifically in CAM-xenografts derived from HT29 cells, but not in HCT116 xenografts, as determined by µPET/MRI. The CAM-grown xenografts were histologically characterised, demonstrating vascularisation of the graft, preserved expression of human CXCR4, and viability of the tumour cells within the grafts. In vivo viability was further confirmed by µPET/MRI measurements using 2-[^18^F]FDG as a surrogate for glucose metabolism. [^68^Ga]Ga-Pentixafor µPET/MRI scans showed distinct radiotracer accumulation in the chick embryonal heart, liver, and kidneys, whereas 2-[^18^F]FDG uptake was predominantly found in the kidneys and joints of the chick embryos. Our findings suggest that [^68^Ga]Ga-Pentixafor is an interesting novel radiotracer for CRC imaging that is worth further investigation. Moreover, this study further supports the suitability of the CAM-xenograft model for the initial preclinical evaluation of targeted radiopharmaceuticals.

## 1. Introduction

Colorectal cancer (CRC) is the second leading cause of cancer-related deaths and the third-most diagnosed cancer worldwide, accounting for 10% of all cancer diagnoses. Although the 5-year survival rate of patients with localised CRC is around 91%, metastasis to distant organs, especially to the liver, dramatically reduces the 5-year survival rate to approximately 15% [1,2,3].

Liver metastases have been linked to an increased expression of the C-X-C chemokine receptor type 4 (CXCR4) [4,5]. CXCR4 is a G-protein-coupled receptor specific to C-X-C motif ligand 12 (CXCL12), a chemokine, abundantly expressed in the liver [6]. It is known that the target organs produce and release specific chemokines that attract cancer cells expressing the corresponding chemokine receptor [6,7]. CXCR4 overexpression is further associated with recurrence and progression of CRC as well as shorter survival of CRC patients [4,8,9]. This makes the CXCR4-CXCL12 axis an interesting therapeutic target [4] and CXCR4 a promising prognostic imaging biomarker.

So far, colonoscopy is the gold standard for CRC diagnosis. During the procedure, biopsy material can be taken to be histopathologically evaluated [10,11]. However, frequent sampling to monitor therapy response or to investigate the dynamics of the tumour is rather inconvenient because of the invasiveness of tissue biopsies. In addition, local sampling may be biased due to inter- and intratumoural heterogeneity [12]. Additional diagnostics often include imaging studies to assess the tumour stage, plan the treatment, and evaluate its effectiveness [13,14]. Nuclear molecular imaging is therefore an important complementary method to histopathological assessment [15,16,17]. Multimodal imaging using positron emission tomography (PET) coupled with magnetic resonance imaging (MRI) or computed tomography provides anatomical information and simultaneously allows for sensitive in vivo investigation of biological processes on the molecular level [18].

The radioactively labelled glucose analogue 2-deoxy-2-[^18^F]fluoro-D-glucose (2-[^18^F]FDG) [19] remains the workhorse of nuclear medicine [20,21] and is the only radiotracer used in routine clinical imaging of CRC to date [17,22]. [^68^Ga]Ga-Pentixafor, a CXCR4-targeting PET tracer with favourable binding characteristics and high metabolic stability, was originally developed for imaging of lymphoproliferative diseases [23,24,25]. To our knowledge, the use of [^68^Ga]Ga-Pentixafor as a diagnostic or prognostic imaging tool for CRC has not yet been reported in a peer-reviewed publication.

As CXCR4 plays a role in CRC prognosis, we propose that [^68^Ga]Ga-Pentixafor may be useful for CRC PET imaging. Therefore, we performed in vitro binding studies with human colon-cancer-derived cell lines. For further evaluation of the tracer’s suitability for CRC imaging, an in vivo xenograft model of intermediate complexity was selected. We established the chorioallantoic membrane (CAM)-xenograft model for CRC imaging using the CXCR4-expressing human cell lines HT29 and HCT116 [26,27,28], as their successful growth on the CAM was previously shown [29,30,31,32].

The use of fertilised chicken eggs (in ovo model) is well-described for tumour biology research [33,34] and also other disciplines [35]. In the last 10 years, the interest of the nuclear imaging community in this model has increased [36]. Imaging studies were performed using different radiotracers, cancer models, and imaging modalities [37,38,39,40,41,42,43,44,45,46]. There are already initial studies showing the comparability of the in ovo model to mice [40,45], suggesting good transferability of results to other animal species. The extra-embryonal CAM is not innervated [47], and the immune system of the chick embryo is not fully developed, until the embryo development day (EDD) 18 [48,49]. This natural immune deficiency and the high vascularisation of the CAM enable the transplantation of cells, organoids, or tissues derived from different species (xenograft) [33,34,50,51,52]. Tumour growth is usually faster compared to rodent xenografts [34] and can be easily monitored due to the accessibility of the CAM [35]. In addition, the in ovo model is a cost-effective alternative to genetically modified mammalian models used in oncology research, as it requires far less effort in terms of breeding, husbandry, and personnel.

In many countries, including Austria, the use of non-mammalian embryos currently does not require ethical approval by competent authorities if these are used before hatching [53]. Experiments with fertilised chicken eggs are therefore an ethically justifiable and socially accepted alternative to mammals in line with the 3Rs of animal testing [54]. The administrative burden for scientists is thereby significantly reduced and the first in vivo studies are thus facilitated.

Here, we report the first in vitro binding studies of [^68^Ga]Ga-Pentixafor to human colon cancer cell lines and the establishment of the CAM-xenograft model for CRC imaging. We performed in ovo PET/MR studies investigating [^68^Ga]Ga-Pentixafor as a novel approach for CRC imaging and using 2-[^18^F]FDG as the current gold standard. Histopathological analyses of CXCR4 and several cancer biomarkers in the harvested xenografts complement the data.

## 2. Materials and Methods

### 2.1. Radiolabelling

Radiosynthesis of [^68^Ga]Ga-Pentixafor was performed on an automatised GAIA V2 synthesis unit (Elysia raytest GmbH, Straubenhardt, Germany) using the SCX reagent kit for labelling of [^68^Ga]-peptides (ABX, Radeberg, Germany) within approximately 30 min. The standard radiolabelling procedure was modified to increase the molar activity and to yield a higher activity concentration, both necessary for preclinical experiments. Briefly, a ^68^Ge/^68^Ga generator (GalliAd; SamNordic, Nacka Strand, Sweden) was eluted with 1.1 mL 0.1 M HCl and the eluate (615 ± 118 MBq) was transferred to a cation exchange cartridge. The cartridge was eluted with 5 M NaCl and added to a solution of 20 or 30 µg PentixaFor acetate (PentixaPharm GmbH, Würzburg, Germany), 2.3 mL ammonium acetate buffer, and 5 µL 1 M ascorbic acid (pH 4.5). The mixture was heated for 7.5 min at 95 °C. The product was immobilised on a Sep-Pak^®^ Plus Light C18 cartridge (Waters Corporation, Milford, MA, USA), washed with sterile water, eluted using 0.5 mL ethanol/water (60/40, *v*/*v*), and formulated in 0.5 mL physiological NaCl. To obtain a well-tolerated final formulation, the ethanol content, which was approximately 33%, was reduced by vacuum concentration for 40 min at 60 °C (Eppendorf^®^ Concentrator; Eppendorf, Hamburg, Germany). The residual ethanol amount was assessed using gas chromatography. Quality control of the obtained radiolabelled peptide consisted of analytical radio high performance liquid chromatography (HPLC) (Chromolith^®^ Performance RP-18e column, 100 × 4.6 mm; 5 µm (Merck KGaA, Darmstadt, Germany), eluents A = 0.1% TFA in water, B = 0.1% TFA in acetonitrile, gradient 0–10 min, 95 to 60% A, flowrate = 2 mL/min) to check the radiochemical purity of the compound and a thin layer chromatography (TLC) using 0.1 M Na–citrate buffer (pH 5) and 1.0 M ammonium acetate/methanol (50/50, *v*/*v*) to detect free ^68^Ga(III) and potential colloids in the product, respectively. In addition, the product was tested for pH and osmolality. [^68^Ga]Ga-Pentixafor in vitro stability was previously reported [55,56]. 2-[^18^F]FDG was prepared for routine diagnostics applying standard procedures in accordance with the state of the art in radiopharmaceutical preparations using a fully automated cassette-based synthesizer (FASTlab^TM^; GE Healthcare, Uppsala, Sweden). All chemicals, unless stated otherwise, were purchased from Merck KGaA (Darmstadt, Germany).

### 2.2. Cell Culture

CXCR4-expressing human colon cancer cell lines HT29 and HCT116 [26,27,28] were obtained from the American Type Culture Collection (Manassas, VA, USA). Cells were kept under standardised conditions in a humidified incubator at 37 °C in an atmosphere containing 95% air and 5% CO_2_. Cells were grown as monolayers in RPMI-1640 medium, supplemented with 2 mM L-glutamine, 100 units/mL penicillin, 100 µg/mL streptomycin, and 10% foetal bovine serum (FBS). All cell culture reagents were purchased from Gibco^TM^, Thermo Fisher Scientific (Waltham, MA, USA).

### 2.3. In Vitro Cell Internalisation Experiments

Cell internalisation experiments were performed as previously described [57], with minor modifications. Two days prior to the experiment, 2.5 × 10^5^ HT29 or HCT116 cells were seeded into 12-well cell culture plates using fully supplemented cell culture medium. Approximately 1 h before the experiment, the medium was replaced by FBS-free cell culture medium and the average number of cells per well was determined using three representative wells per cell line; 5 pmol [^68^Ga]Ga-Pentixafor (corresponding to 60.4 ± 28.8 kBq) was added to each well (10 nM final concentration per well). To determine the specificity of [^68^Ga]Ga-Pentixafor binding towards CXCR4, cells were co-incubated with either a 1000-fold excess of CXCR4 Antagonist III (Calbiochem^®^, Merck KGaA, Darmstadt, Germany) or with bi-distilled water as vehicle control. After 1 h of radioligand incubation, the supernatant was collected and the cells were washed twice with 1 mL ice-cold Dulbecco’s phosphate buffered saline, yielding the free radiotracer fraction. Cells were then incubated twice with 1 mL of ice-cold acidic glycine solution (100 mM NaCl, 50 mM glycine, pH 2.8) for 5 min to recover the membrane-bound fraction of [^68^Ga]Ga-Pentixafor. Cells were lysed using 1 mL of 1 M NaOH to collect the internalised fraction. Finally, wells were washed twice with 1 mL 1 M NaOH and wash fractions were combined with the internalised fraction. All obtained radioactive fractions were gamma-counted (Wizzard^2®^ 3” Gamma Counter 2480; PerkinElmer, Waltham, MA, USA). Additionally, three aliquots of [^68^Ga]Ga-Pentixafor (5 pmol each) were counted as a reference to determine the applied dose (AD) in counts per minute (CPM). Specific fractions of membrane binding and internalisation were calculated by subtracting unspecific CPM from total CPM for every single well. Mean %AD/well was calculated out of triplicates and normalised to 1 × 10^6^ cells.

### 2.4. In Ovo Assay and Tumour Cell Inoculation

Procedures for incubating fertilised chicken eggs and tumour cell inoculation followed previously published protocols [39,42,45,58,59,60] with some modifications. In brief, specific pathogen-free eggs (premium plus) were purchased from Charles River Laboratories (Wilmington, MA, USA) and delivered via cooling transport. Fertilised chicken eggs were either immediately incubated or stored at 4 °C for up to 7 days upon arrival. After acclimatisation at room temperature, the eggshell was cleaned using 70% ethanol. Eggs were then incubated horizontally under humidified atmosphere (60–65% relative humidity) at 37 °C starting at EDD1. At EDD3, the eggs were rotated 180° horizontally and carefully pierced using a sterile needle (B. Braun Melsungen AG, Melsungen, Germany). The small hole was then covered using semi-permeable tape (Leukosilk^®^ BSN medical GmbH, Hamburg, Germany) to protect it from contamination. On EDD5, after the CAM has lowered, the hole was enlarged using tweezers and scissors. For the generation of CAM-grown xenografts, 3 × 10^6^ cells were mixed with 20 µL extracellular matrix (Matrigel^®^, high-concentrated; Corning, NY, USA) and inoculated onto the CAM on EDD9. To this, a silicone O-ring (MVQ material, 6 × 0.5 mm; Arcus, Hamburg, Germany) was placed on the CAM at the junction of two vessels or at the end of a capillary after slight laceration with cotton swabs to ensure rapid vascularisation. Embryonal vitality and tumour growth were monitored daily by visual inspection. All experimental procedures were completed on EDD19 at the latest. The tumours were harvested between EDD15-EDD19 and preserved for further histopathological evaluation.

### 2.5. PET/MR Imaging

PET and MR were measured simultaneously using a preclinical MRI scanner (BioSpec^®^ 94/30 with 9.4 Tesla; Bruker Biospin, Ettlingen, Germany) with a dedicated PET insert (model Si 168; Bruker Biospin, Ettlingen, Germany) and an 86 mm PET-compatible coil (model T20202V3; Bruker Biospin, Ettlingen, Germany). Paravision 360 V3.2 software (Bruker Biospin, Ettlingen, Germany) was used for data acquisition. For both CAM-xenograft models (HT29 and HCT116), [^68^Ga]Ga-Pentixafor baseline (EDD15-EDD18) and blocking scans (EDD17/18) were performed. In addition, PET/MRI measurements were performed with 2-[^18^F]FDG on EDD18/19. When possible, the measurements were carried out as part of a longitudinal study. For a detailed overview of measurements refer to Table S1.

#### 2.5.1. Image Acquisition

On the day of imaging, 10.9 ± 6.27 MBq (2.4 ± 1.2 nmol peptide) [^68^Ga]Ga-Pentixafor was injected into a CAM-vessel using a 30 G sterile needle and LDPE tubing (length 20 cm, inner diameter: 0.28 mm, outer diameter: 0.61 mm). The catheter was pre-flushed with physiological saline to ensure intravascular (i.v.) positioning of the needle before tracer injection. For CXCR4 blocking experiments, 315 µg AMD3100 (CXCR4 Antagonist I) (Calbiochem^®^, Merck KGaA, Darmstadt, Germany) in 50 µL sterile water was injected immediately before tracer application. Based on an embryonal body weight of 21 g on EDD18 [61], the dose corresponds to ~15 mg/kg body weight. Accordingly, for 2-[^18^F]FDG scans, 5.84 ± 2.27 MBq of 2-[^18^F]FDG was injected into a CAM-vessel. For both radiotracers, the total injection volumes never exceeded 200 µL.

After radiotracer injection, the chicken egg was placed in the incubator to allow the radiotracer to distribute for 20 min at 37 °C. The chick embryo was anaesthetised for 6 min using 3% isoflurane in oxygen (flow rate 2 mL/min) as previously shown to be best-suited for both single and repeated in ovo PET imaging [62]. The egg was placed into a custom 3D-printed holder and anaesthesia was maintained using 2% isoflurane in oxygen. MR measurements were immediately started, and a static PET scan (15 min) was started 61 ± 4.7 min post injection (p.i.).

The following MRI sequences were acquired: a 3D T1-weighted iso-voxel fast low-angle shot (FLASH) sequence covering the whole egg in isotropic resolution (repetition time (TR): 15 ms, echo time (TE): 3.625 ms, matrix size (MS): 180 × 180 × 300, field of view (FOV): 90 × 90 × 150 mm^3^, voxel size: 0.5 mm isotropic, acquisition time (AT): 13 min 30 s) and a T2-weighted rapid acquisition with relaxation enhancement (TurboRARE) sequence (coronal: TR/TE: 12500/40.92 ms, slice thickness (SL): 0.5 mm, number of slices (NS): 82, MS: 388 × 278, FOV: 70 × 50 mm^2^, pixel size: 0.18 × 0.18 mm, rare factor: 8, AT: 7 min 5 s; axial: TR/TE: 15,000/40.92 ms, SL: 0.5 mm, NS: 125, MS: 388 × 250, FOV: 70 × 45 mm^2^, pixel size: 0.18 × 0.18 mm, rare factor: 8, AT: 7 min 45 s). Attenuation maps were generated based on the T1-weighted FLASH sequence.

#### 2.5.2. Image Post-Processing and Data Analysis

The PET data were attenuation-corrected for the egg itself, the used cradle, and the MR coil. Furthermore, the PET data were corrected for scatter, deadtime, and random coincidences. List-mode data were reconstructed using the ordered subset expectation maximisation (OSEM) algorithm with 16 subsets, a pixel size of 0.5 × 0.5 mm, and an MS of 180 × 180. Image datasets were further analysed using the PMOD software version 3.807 (PMOD Technologies, Zürich, Switzerland). Volumes of interest (VOIs) were identified based on MR images (T2-weighted TurboRARE sequence) (Figure S1) and manually delineated. A sphere was placed around the whole egg to extract the total injected activity (TA). For a subpopulation of subjects bearing HCT116 xenograft imaged with [^68^Ga]Ga-Pentixafor (*n* = 4) and for six subjects imaged with 2-[^18^F]FDG, the following organs were delineated: brain, eyes, liver, heart, gizzard, lungs, and kidneys. Furthermore, to assess the background radioactivity, a sphere (2 mm radius) was placed in egg yolk and egg albumen.

PET and MR data were fused using the rigid matching function of PMOD. Image fusion was manually adjusted, if necessary. The MR-based VOIs were transferred to the fused data. Joints (intertarsal) and bones (tarsometatarsal) were delineated manually based on the fused PET/MR data. Examples of delineation and positioning of the VOIs for xenografts and organs are depicted in Figures S2 and S3. The exported VOI statistics were manually decay corrected for the time of PET start using Excel, and the percentage of total activity per volume (%TA/cc) was calculated.

### 2.6. Histopathological Evaluation of the In Ovo CAM Xenografts

Harvested xenografts were fixed overnight in 4% paraformaldehyde (PFA) in phosphate-buffered saline (PBS) pH 6.8 (Morphisto, Offenbach am Main, Germany) and stored in 0.1% PFA in PBS for up to 14 days before further processing. Specimens were dehydrated and embedded in paraffin, and 2-µm-thick sections were prepared using a rotary microtome (Epredia^TM^ HM 340 E; Thermo Fisher Scientific, Waltham, MA, USA). Sections were left to dry overnight and then incubated for 30 min at 57 °C to melt the paraffin. Paraffin was removed by incubation in xylene (3 × 10 min) and the sections were rehydrated.

For haematoxylin–eosin staining (H&E) (*n* ≥ 9 per cell line), cell nuclei were stained with undiluted haematoxylin (Morphisto, Offenbach am Main, Germany) for 1 min 40 s. To stain the cytoplasm of the cells, sections were incubated with 0.5% eosin solution for 1 min 20 s. Slides were dehydrated and mounted using VectaMount^®^ Permanent Mounting Medium (Vector Laboratories, Newark, NJ, USA).

For immunohistochemical (IHC) analyses (*n* ≥ 3 per marker and cell line), antigen retrieval was performed using citrate buffer (pH 6.0) (Abcam, Cambridge, UK) in a microwave for 2 min at 1000 Watts, followed by 10 min at 100 Watts. To block the endogenous peroxidase activity, sections were incubated with 3% H_2_O_2_ for 10 min. Sections were subsequently blocked using avidin and biotin (Avidin/Biotin Blocking Kit; Vector Laboratories, Newark, NJ, USA). When detecting an endogenous target, cell membranes were permeabilised using 0.2% Tween 20 before blocking with Bloxall^®^ Endogenous Blocking Solution (Vector Laboratories, Newark, NJ, USA). Sections were incubated with primary antibodies overnight at 4 °C as follows: antibody against desmin (1:100, clone 33, no.: M0760; Dako, Agilent, Santa Clara, CA, USA), cytokeratin 19 (1:100, clone RCK108, no.: M0888; Agilent, Santa Clara, CA, USA), Ki67 (1:200, clone SP6, no.: ab16667; Abcam, Cambridge, UK), cleaved caspase 3 (1:2000, clone 5A1E, no.: #9664; Cell Signaling Technology, Danvers, MA, USA), and CXCR4 (1:500, clone UMB2, no.: ab12824; Abcam, Cambridge, UK). The next day, sections were incubated with a suitable biotinylated secondary antibody (Vector Laboratories, Newark, CA, USA) for 1–2 h. Signal detection was based on the avidin–biotin complex (ABC) and 3,3′-diaminobenzidin tetrahydrochloride (DAB) using dedicated reagent kits according to the manufacturer’s instructions (Vectastain^®^ ABC Kit; Vector Laboratories, Newark, NJ, USA; DAB Substrate Kit; Abcam, Cambridge, UK). The cell nuclei were counterstained using undiluted haematoxylin for 1.5 min. After dehydration, slides were mounted using VectaMount^®^ Permanent Mounting Medium. Images of the stained sections were captured using a brightfield microscope (BX53; Olympus, Tokyo, Japan) and 4×, 10×, or 20× objective magnification.

### 2.7. Statistical Analyses

Statistical analyses were performed, and graphs were prepared using GraphPad Prism version 8.2.1 (GraphPad Software, Inc., San Diego, CA, USA). Appropriate statistical tests for paired (in vitro baseline vs. blocking, paired *t*-test or Wilcoxon matched-pairs signed rank test, if not normally distributed) and unpaired (comparison of uptake between the two cell lines, in ovo data, unpaired *t*-test or Mann–Whitney Test, if not normally distributed) datasets were used. For HCT116 xenografts, an additional paired *t*-test was performed for eight of the nine subjects, as for these datasets, baseline and blocking scans were performed in the same individual. A *p*-value < 0.05 was assumed to be statistically significant. Given values present mean ± SD, if not stated otherwise.

## 3. Results

### 3.1. Radiolabelling

The modified radiolabelling procedure yielded, after vacuum concentration, 183 ± 46 MBq [^68^Ga]Ga-Pentixafor (*n* = 15). Decay corrected radiochemical yield was 60 ± 11% in radiochemical purity greater than 95% (Figures S4 and S5). The mean specific activity was 7.03 ± 1.04 MBq/μg peptide, and the mean activity concentration was 370 ± 9 MBq/mL at the end of synthesis. Furthermore, ethanol content (33%) was reduced to less than 0.1% in the final product, as determined by gas chromatography.

### 3.2. In Vitro Cell Internalisation Experiments

**Figure 1:**
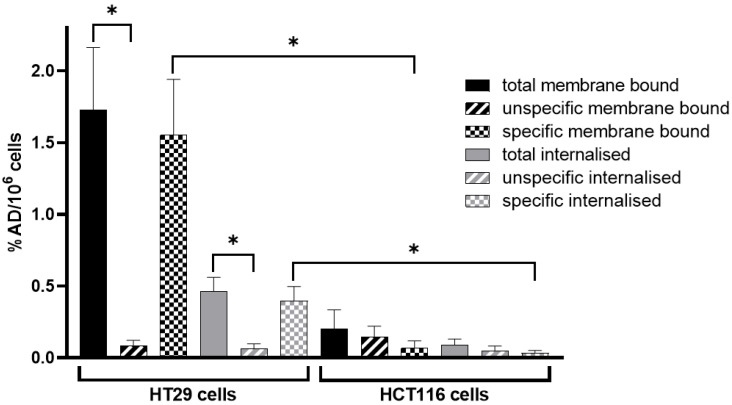
Total, unspecific, and specific membrane binding and internalisation of [^68^Ga]Ga-Pentixafor in HT29 and HCT116 cells are shown. At least three independent experiments were performed in triplicate. Data are plotted as % applied dose normalised to 1 × 10^6^ cells (%AD/10^6^ cells) and presented as mean ± SD. Datasets that are significantly different (*p* < 0.05) are marked with an asterisk (*).

Specific uptake (membrane binding and internalisation) of [^68^Ga]Ga-Pentixafor was significantly higher in HT29 than in HCT116 cells (Figure 1). The membrane-bound fraction of [^68^Ga]Ga-Pentixafor was low in HCT116 cells and internalisation was almost negligible. Co-incubation with 1000-fold excess of competitor (AMD3100) did not lead to a significantly reduced radioactive signal in HCT116 cellular fractions (*n* = 3 in triplicates). Membrane-bound and internalised fractions of [^68^Ga]Ga-Pentixafor were highly specific regarding HT29 cells (*n* = 6 in triplicates). Exact uptake values can be found in Table S2.

### 3.3. Multimodal Imaging

#### 3.3.1. PET/MRI Measurements Using [^68^Ga]Ga-Pentixafor

**Figure 2:**
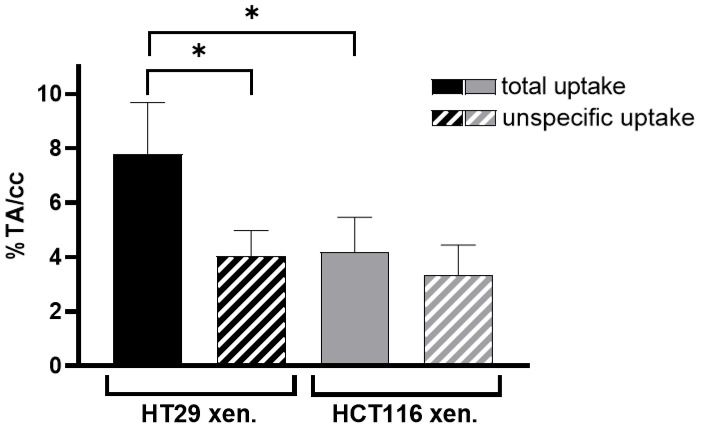
Total and unspecific uptake of [^68^Ga]Ga-Pentixafor in HT29 CAM-xenografts and HCT116 CAM-xenografts. Unspecific uptake was determined by co-injection of an excess of AMD3100 (blocking condition). Experiments were performed using at least three different individuals (*n* ≥ 3). For HCT116 CAM-xenografts, 8 paired datasets were obtained (baseline: *n* = 9, blocking: *n* = 8). For HT29 CAM-xenografts, longitudinal scanning was possible only with two subjects (baseline: *n* = 7, blocking: *n* = 3). The use of paired and unpaired datasets was considered in the statistical analysis. The total number of counts in the egg (extracted from a spherical VOI covering the whole subject) was taken as the total activity. Data are plotted as percentage of total activity per cm^3^ (%TA/cc) and presented as mean ± SD. Datasets that are significantly different (*p* < 0.05) are marked with an asterisk (*).

**Figure 3:**
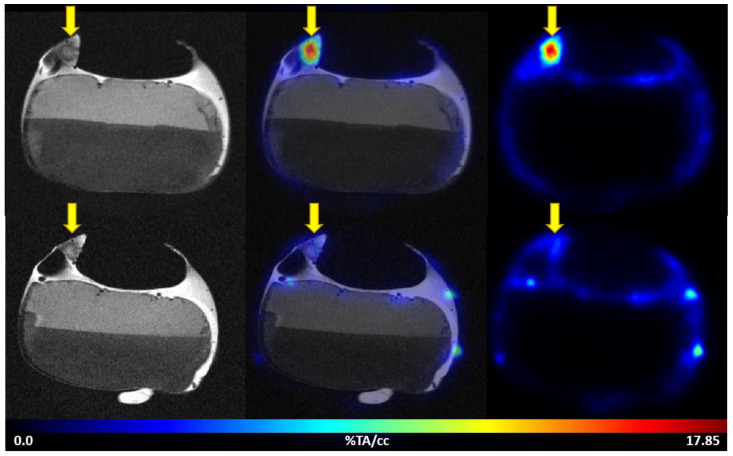
Representative images of [^68^Ga]Ga-Pentixafor PET/MRI in the HT29 CAM-xenograft model. Upper row: baseline scan after i.v. injection of 3.50 MBq [^68^Ga]Ga-Pentixafor (0.47 nmol peptide) on EDD15. Lower row: blocking (315 µg AMD3100/egg) of [^68^Ga]Ga-Pentixafor uptake (7.57 MBq, 0.93 nmol peptide) in the same subject on the following day (EDD16). Left: T2-weighted TurboRARE images, right: [^68^Ga]Ga-Pentixafor PET 60 min p.i., middle: fused PET/MRI (axial planes are shown). HT29 CAM-xenografts are marked with a yellow arrow.

**Figure 4:**
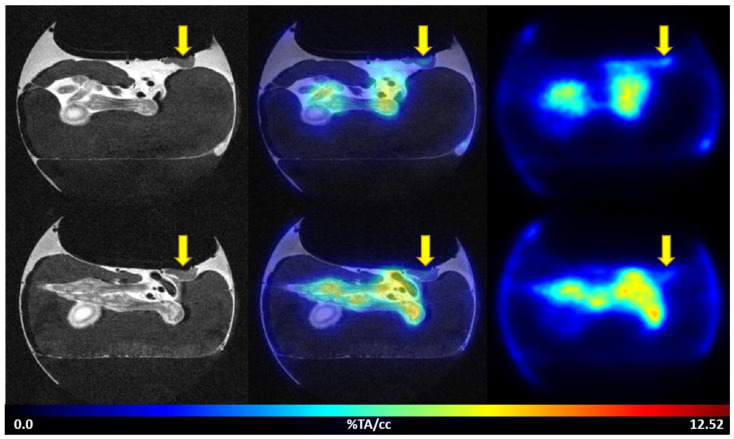
Representative images of [^68^Ga]Ga-Pentixafor PET/MRI in the HCT116 CAM-xenograft model. Upper row: baseline scan after i.v. injection of 7.19 MBq [^68^Ga]Ga-Pentixafor (0.79 nmol peptide) on EDD16. Lower row: blocking (315 µg AMD3100/egg) of [^68^Ga]Ga-Pentixafor uptake (18.32 MBq, 3.12 nmol peptide) in the same subject on the following day (EDD17). Left: T2-weighted TurboRARE images, right: [^68^Ga]Ga-Pentixafor PET 60 min p.i., middle: fused PET/MRI (axial planes are shown). HCT116 CAM-xenografts are marked with a yellow arrow.

The uptake of [^68^Ga]Ga-Pentixafor was investigated under baseline and blocking conditions in two CAM-xenograft models of CRC (HT29 and HCT116) (Figure 2, Table S3). A significantly higher accumulation of [^68^Ga]Ga-Pentixafor was found in CAM-xenografts derived from HT29 cells (7.80 ± 1.88%TA/cc, *n* = 7) than in CAM-xenografts derived from HCT116 cells (4.17 ± 1.29%TA/cc, *n* = 9). Administration of the blocking agent AMD3100 significantly reduced [^68^Ga]Ga-Pentixafor uptake in HT29-derived CAM-xenografts to 4.05 ± 0.92%TA/cc (*n* = 3, *p* < 0.05). Specific binding was relatively low in HCT116 xenografts (<1%TA/cc) and significant differences between baseline and blocking measurements could only be shown when referring to the same subject (paired *t*-test, *p* < 0.05, *n* = 8). Representative [^68^Ga]Ga-Pentixafor PET/MR images of the HT29 and HCT116 CAM-xenograft models are shown in Figure 3 and Figure 4, respectively.

Based on the T2-weighted TurboRARE images, xenografts and organs were identified and manually delineated (Figures S1–S3). The highest accumulation of [^68^Ga]Ga-Pentixafor was found in the heart, followed by the liver and kidneys. However, the uptake was not reduced in these organs under blocking conditions (Table S4, Figures S6 and S7).

#### 3.3.2. PET/MRI Measurements Using 2-[^18^F]FDG

**Figure 5:**
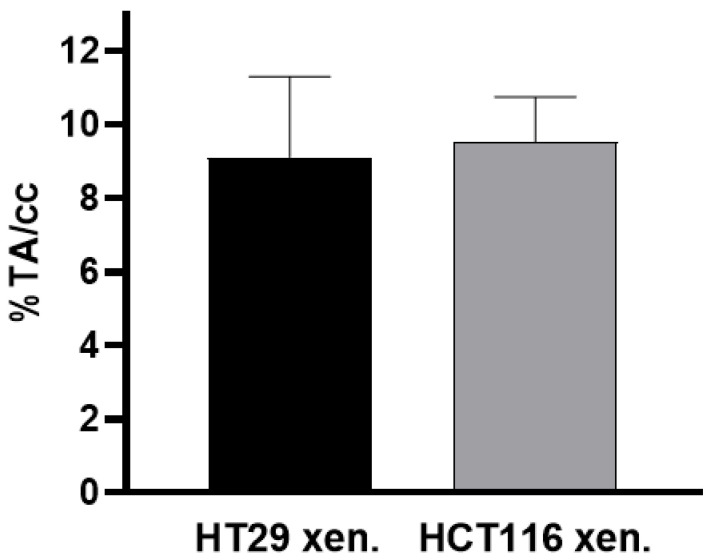
Accumulation of 2-[^18^F]FDG in xenografts derived from HT29 and HCT116 cells. Data are depicted as %TA/cc and presented as mean ± SD (*n* ≥ 3 per xenograft model).

**Figure 6:**
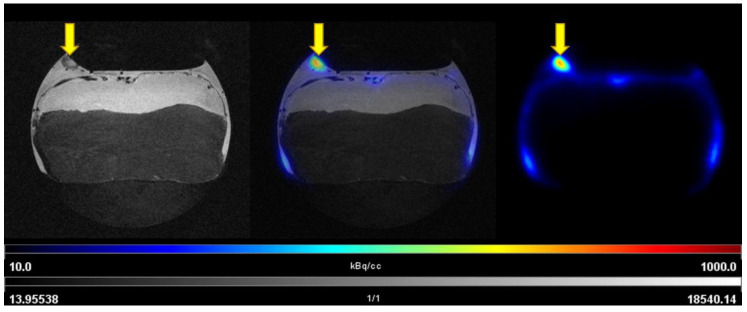
Representative images of 2-[^18^F]FDG PET/MRI on EDD18 using the HT29 CAM-xenograft model. PET imaging was performed 60 min after injection of 8.94 MBq 2-[^18^F]FDG into a CAM-vessel on EDD18. Left: T2-weighted TurboRARE image, right: PET, middle: fused PET and MR (axial planes are shown).

**Figure 7:**
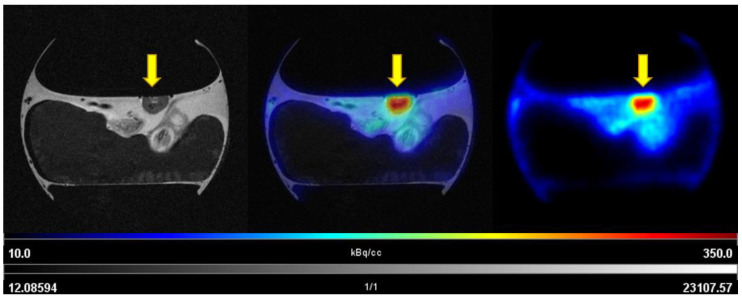
Representative images of 2-[^18^F]FDG uptake into HCT116 CAM-xenograft. Uptake of 2-[^18^F]FDG into the HCT116 xenograft after injection of 4.21 MBq 2-[^18^F]FDG into a CAM-vessel on EDD18. PET imaging was performed 60 min p.i. Left: T2-weighted TurboRARE image, right: PET, middle: fused PET and MR (axial planes are shown).

2-[^18^F]FDG was avidly taken up into CAM-xenografts derived from both colon cancer cell lines: the extent of 2-[^18^F]FDG uptake was comparable in HT29 CAM-xenografts (9.12 ± 2.19%TA/cc, *n* = 3) and HCT116-derived xenografts (9.53 ± 1.22%TA/cc, *n* = 4) (Figure 5). Representative images of 2-[^18^F]FDG PET/MRI for both CAM-xenograft models are shown in Figure 6 and Figure 7, respectively. Also, 2-[^18^F]FDG uptake was found in the joints of the chick embryo. High uptake of the radiolabelled glucose analogue was further observed in the kidneys followed by the liver and the brain (Table S5, Figure S8). Interestingly, the extent of joint uptake seemed to be depended on the time of day of imaging (Figures S9 and S10).

### 3.4. Histopathological Evaluation of the In Ovo CAM-Xenografts

**Figure 8:**
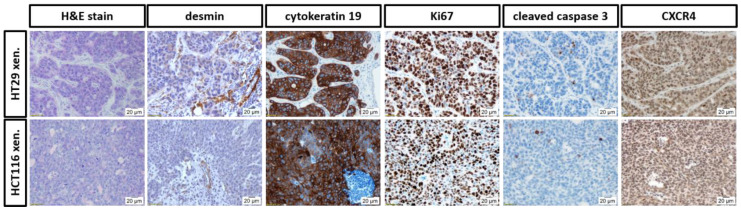
Characterisation of HT29 (upper row) and HCT116 (lower row) CAM-xenografts. Representative pictures of different stainings performed on CAM-xenografts. From left to right: H&E stain; anti-desmin staining (pericytes in the vascular network); anti-cytokeratin 19 staining (epithelial cells); anti-Ki67 staining (proliferating cells); anti-cleaved caspase 3 staining (apoptotic cells) and anti-CXCR4 staining (target of interest). Magnification: 200-fold. At least three sections from different specimens were stained per cancer cell line and tumour marker.

CAM-xenografts derived from HT29 and HCT116 cells were used for H&E staining and IHC (Figure 8). H&E staining allowed for information regarding the histomorphology of the specimens and the density of the tumour cells within the graft. HCT116 xenografts showed a densely packed tumour cell mass with clear tumour margins. On the other hand, HT29 cells grew in organised tumour cell groups. CAM connective tissue and residues of the basal matrix (Matrigel^®^, high-concentrated; Corning, NY, USA) were identified in some samples between tumour cells or within tumour cell groups. In the H&E staining, blood vessels were predominantly observed at the tumour rim. Small capillaries were found also within the xenograft. IHC analyses using an antibody against desmin demonstrated vascularisation of the grafts derived from both cell lines. Cytokeratin 19 is a marker for cells of epithelial origin and was used to differentiate between the inoculated cancer cells and other parts of the graft, such as CAM, blood vessels, or immune infiltrates. The majority of the tumour cells within the HT29 and HCT116 xenografts were viable, as shown using an antibody against Ki67. On the other hand, only isolated cells were apoptotic as obvious from cleaved caspase 3 staining. Generally, xenografts derived from both cell lines were positive for CXCR4. CXCR4 expression was detected in the cytoplasm and for HT29 xenografts also in nuclei.

## 4. Discussion

CXCR4 is the most conserved chemokine receptor across species and is expressed by a variety of cell types [63,64]. However, there is a high cell-type-dependent heterogeneity that is probably caused by post-translational changes [63]. Mouse and human CXCR4 proteins are 90% identical, while chicken CXCR4 is 82% identical to human CXCR4 protein [64]. The affinity of [^nat^Ga]Ga-Pentixafor for mouse CXCR4 is IC_50_ > 1000 nM, while it is approximately 25 nM for the human equivalent as assessed by displacement studies using [^125^I]CPCR4.3 or [^125^I]FC-131, respectively [65]. The affinity of [^nat^Ga]Ga-Pentixafor for the chicken CXCR4 is yet unknown. However, it was shown that chicken CXCR4 responds to the human CXCL12 [64].

Within this study, in vitro binding studies using two human colon cancer cell lines were performed. Membrane binding and internalisation of [^68^Ga]Ga-Pentixafor was high in HT29, but unexpectedly low in HCT116 cells. Specificity of membrane binding and internalisation was shown in HT29 cells, but not in HCT116 cells. This finding was somewhat contradictory to studies in the literature reporting high CXCR4 expression for HCT116 cells determined by western blotting [26]. The performed IHC staining against human CXCR4 of the obtained CAM-xenografts also indicated CXCR4 expression in HCT116-xenograft-derived specimens. For both cell lines, the staining was predominantly cytoplasmic, whereas nuclear staining was additionally detected for HT29 xenografts (Figure 8, descriptive analysis).

Apparently, there is a discrepancy in CXCR4 antibody binding and radioligand binding, which may result from the different treatment of samples and experimental procedures: reduced or denatured proteins are used for western blotting, while radioligand binding assays are performed on native proteins as intact binding pockets are required. CXCR4 proteins expressed by HCT116 may be not (fully) functional—a fact that would be overlooked using techniques not based on native proteins. Furthermore, CXCR4 possesses two non-identical, neighbouring binding sites [66]. Seven known splice variants exist for CXCR4 [67], and the same three of these have been reported for the used cell lines [68,69]. The binding affinities of [^68^Ga]Ga-Pentixafor towards the different splice variants are undetermined. For two of the mentioned splice variants, two pathogenic mutations are described for HCT116 cells, that are not reported for HT29 cells. One of these mutations is a silent mutation, whereas the other causes an amino acid change [68,69,70,71]. Unfortunately, it has not been investigated, whether this mutation affects [^68^Ga]Ga-Pentixafor binding. A detailed investigation of the potential non-identical CXCR4 proteins expressed by HCT116 and HT29 cells will be undertaken but is out of the scope of the current manuscript.

To assess the tracer’s suitability for in vivo imaging, uptake of [^68^Ga]Ga-Pentixafor was further investigated using two different CAM-xenograft models of CRC (HT29 and HCT116). The outcome of the in ovo imaging study essentially reflects what has been shown in vitro before: [^68^Ga]Ga-Pentixafor accumulation was significantly higher in CAM-xenografts derived from HT29 cells than from HCT116 cells. The uptake of [^68^Ga]Ga-Pentixafor in CAM-xenograft tissues proved to be specific in HT29-derived grafts. Specific uptake of [^68^Ga]Ga-Pentixafor was much lower in CAM-xenografts derived from HCT116 cells.

In addition to [^68^Ga]Ga-Pentixafor, we investigated the uptake of 2-[^18^F]FDG in the two different CAM-xenograft models of CRC. These measurements essentially served as a control group to prove adequate vascularisation and viability (using glucose consumption as a surrogate) of the CAM-xenografts. 2-[^18^F]FDG strongly accumulated in CAM-xenograft tissues, regardless of the human colon cancer cell line from which the grafts were derived. We therefore conclude that the low uptake of [^68^Ga]Ga-Pentixafor in HCT116-derived CAM-xenografts does not result from insufficient vascularisation or poor viability. Our data indicate that CXCR4 imaging using [^68^Ga]Ga-Pentixafor may be of additional value alongside imaging of glucose consumption using 2-[^18^F]FDG PET.

The excellent soft-tissue contrast of the used imaging modality allowed for the delineation of several chick embryonal organs and the subsequent assessment of radiotracer distribution. However, due to the small volumes of embryonal organs and the limited resolution of PET (especially for radionuclides with a relatively high positron range such as gallium-68), image-derived quantification is challenging, and spill-over effects cannot be excluded. In addition, embryonal organ development progresses steadily with continuous incubation of the fertilised eggs. The experimental set-up was designed in such a way that the day(s) of measurement and the embryonal development was as standardised as possible, but a 1–2 day difference in embryonal development could not be avoided. Due to the longitudinal study design, organ harvesting and gamma-counting were not performed after single PET measurements.

Nevertheless, for [^68^Ga]Ga-Pentixafor, we observed similar distribution patterns as previously described for mice: [^68^Ga]Ga-Pentixafor accumulated especially in the adrenal glands, kidneys, liver, and lungs. However, specific organ uptake could not be proven using dedicated blocking agents, which is probably due to the low affinity towards the mouse CXCR4 [23,24,25]. In this in ovo study, [^68^Ga]Ga-Pentixafor accumulated mainly in the liver, heart, kidneys, and lungs (adrenal glands were not identified). The increased accumulation of [^68^Ga]Ga-Pentixafor in the heart, liver, and kidneys is most likely due to their strong blood supply. High uptake of 2-[^18^F]FDG in the chick embryonal joints was previously observed and described to reflect high metabolic activity due to embryonal growth [38,39]. In this study, 2-[^18^F]FDG uptake in the joints was significantly more pronounced when imaging was performed later on the day (Figures S9 and S10); 2-[^18^F]FDG was used for i.v. CAM-injections up to 6 h after radiopharmaceutical production. The quality of the radiopharmaceutical preparation of 2-[^18^F]FDG was re-checked after 6 h, confirming that no relevant amounts of [^18^F]fluoride (<limit of detection) or other impurities were present. Different blood glucose concentrations were reported for chick embryos and blood glucose levels seem to vary when eggs are handled differently [72,73]. In addition, 2-[^18^F]FDG uptake pattern may vary due to other biological variables such as metabolic state or development stage of the chick embryo, which might also be time-of-day-dependent (circadian rhythm). Therefore, standardisation of experimental procedures seem to be of particular importance when using the in ovo model for preclinical imaging studies.

Generally, due to the given time frame (EDD9-19), imaging studies can only be carried out in a limited time window. This also hampers the use of slowly growing tumour cell lines [34,45]. In addition, one needs to consider that metabolic processes may differ between adult rodents and developing chick embryos, but also between different development stages [45]. Again, standardisation of the experimental procedures including the day of measurement with respect to the embryo development stage is of utmost importance. However, our work and the previous work of others clearly demonstrate the strength of the in ovo model for the initial evaluation of radiopharmaceuticals and the preselection of candidate molecules at an early stage of tracer development. In our opinion, the in ovo model represents an in vivo model of sufficient complexity to assess straightforward research questions such as tumour uptake.

## 5. Conclusions

[^68^Ga]Ga-Pentixafor specifically accumulated in the CAM-xenograft model of CRC. The obtained in ovo imaging data match the findings from the in vitro binding study. We conclude that the tracer shows potential for CRC imaging and that this approach should be further investigated in more advanced preclinical models. In addition, this study further supports the applicability of the in ovo CAM-xenograft model for the initial preclinical evaluation of targeted radiopharmaceuticals as already shown by others.

## Data Availability

The used data, additional to those in the supplement, are available from the corresponding author upon a reasonable request.

## Supplementary Materials

The following supporting information can be downloaded at: https://www.mdpi.com/article/10.3390/cancers14225549/s1, Figure S1. T2-weighted TurboRARE MR images of the chick embryo on EDD18; Figure S2. Example for VOI positioning for CAM-xenograft; Figure S3. Example for VOI positioning for different organs and other tissues within the chicken egg; Figure S4. Representative radio-HPLC chromatogram of [^68^Ga]Ga-PentixaFor (red) with the co-injected standard [^nat^Ga]Ga-Pentixafor (blue); Figure S5. Representative TLC-chromatogram of [^68^Ga]Ga-Pentixafor using 0.1 M Na-citrate (left) and 1.0 M ammonium acetate/methanol (right) as solvent; Figure S6. Distribution of [^68^Ga]Ga-Pentixafor under baseline (filled bars) and blocking (bars with pattern) conditions; Figure S7. Representative images of [^68^Ga]Ga-Pentixafor distribution in the chick embryonal organism; Figure S8. 2-[^18^F]FDG distribution in chick embryonal organs, albumen, and yolk; Figure S9. Selected PET/MR images of 2-[^18^F]FDG uptake in two different subjects; Figure S10. Comparison of 2-[^18^F]FDG uptake into different organs and tissues of the chick embryo when performing the scan early (up to 2.5 h after radiolabelling, filled bars) or later (more than 5.5 h after radiolabelling, bars with pattern) during the day; Table S1. Overview on performed scans and subjects; Table S2. Uptake of [^68^Ga]Ga-Pentixafor in HT29 and HCT116 cells; Table S3. Uptake of [^68^Ga]Ga-Pentixafor in CAM-xenografts derived from HT29 and HCT116 cells; Table S4. Accumulation of [^68^Ga]Ga-Pentixafor in organs and other tissues within the chicken egg; Table S5. Accumulation of 2-[^18^F]FDG in embryonal organs as well as HT29 and HCT116 CAM-xenografts.
