# Supplementary material for: CAM-Xenograft Model Provides Preclinical Evidence for the Applicability of [68Ga]Ga-Pentixafor in CRC Imaging
Source: Cancers (Basel). 2022 Nov 11;14(22):5549. doi: 10.3390/cancers14225549 (PMC9688097; doi:10.3390/cancers14225549)
Supplement: Supplementary file 1 [file cancers-14-05549-s001.zip › cancers-1964340-supplementary.pdf]

Supplementary Materials

Table S1. Overview on performed scans and subjects

egg-ID	CAM-xenograft model	[^{68}Ga]Ga-Pentixafor baseline				[^{68}Ga]Ga-Pentixafor blocking				2-[^{18}F]FDG			
		EDD	PET-scan start [min p.i.]	injected peptide [nmol]	tumour uptake [%TA/cc]	EDD	PET-scan start [min p.i.]	injected peptide [nmol]	tumour uptake [%TA/cc]	EDD	PET-scan start [min p.i.]	IA [MBq]	tumour uptake [%TA/cc]
06.22_02	HT29	18	60	2.32	7.98								
06.22_03		16	72	2.82	6.74	17	60	2.70	5.11				
06.22_07		15	60	0.47	11.26	16	60	0.93	3.57				
06.22_13		16	60	3.81	5.09								
06.22_25		16	60	1.42	8.30								
06.22_28		17	60	2.39	8.07								
06.22_50		16	60	1.83	7.13								
06.22_55						16	60	2.10	3.47				
06.22_14										18	60	5.31	10.0
06.22_34										18	60	8.30	6.62
06.22_41										18	60	8.94	10.7
06.22_20	HCT116	17	60	2.60	3.13								
06.22_23		16	60	3.40	3.19	17	60	1.09	2.92				
06.22_29		15	60	1.85	5.06	16	60	0.94	2.48				
06.22_46		16	60	0.79	3.31	17	60	3.12	2.90	18	60	5.12	9.02
06.22_49		18	60	1.94	3.23	16	60	3.28	2.19	19	60	6.50	8.17
06.22_54		16	60	2.79	5.48	17	82	2.85	3.41	18	73	2.48	11.0
06.22_66		16	60	4.05	3.84	17	62	2.60	3.59				
06.22_70		16	60	2.46	6.76	17	60	1.75	5.76	18	60	4.21	9.89
06.22_73		16	60	2.88	3.57	17	60	6.36	3.50				

Identifier (ID)

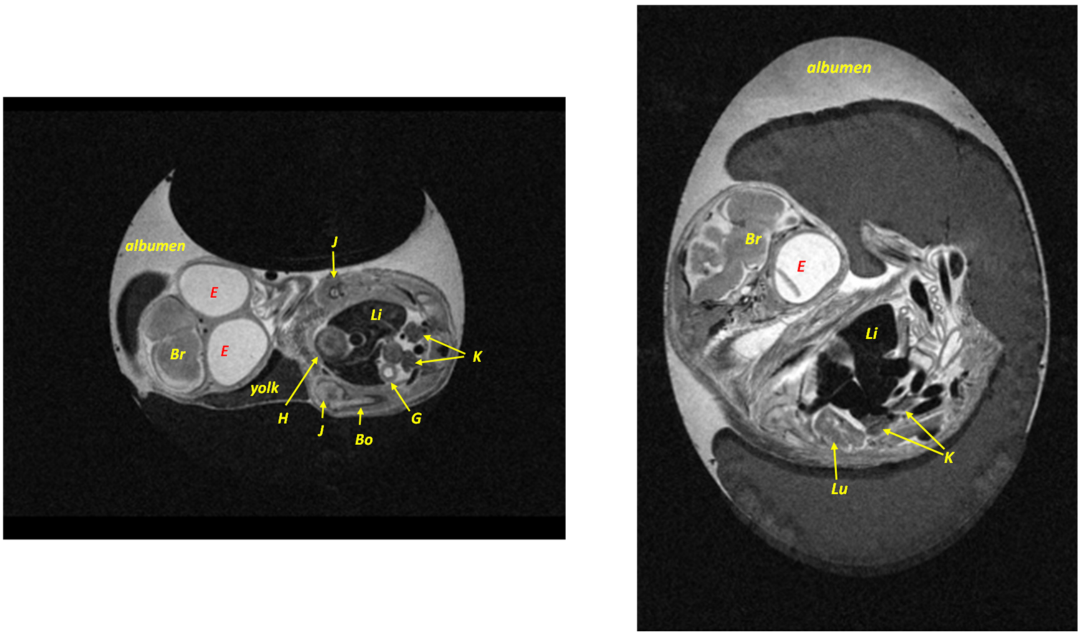

(**a**) (**b**)

Figure S1. T2-weighted TurboRARE MR images of the chick embryo on EDD18: **(a)** axial plane and **(b)** coronal plane. Organs to be delineated and other tissues of the chicken egg are stated or marked with a yellow arrow: Bo (bones – tarsometatarsus), Br (brain), E (eye), G (gizzard), H (heart), J (joints - intertarsal), K (kidneys), Li (liver), Lu (lungs), as well as albumen and yolk.

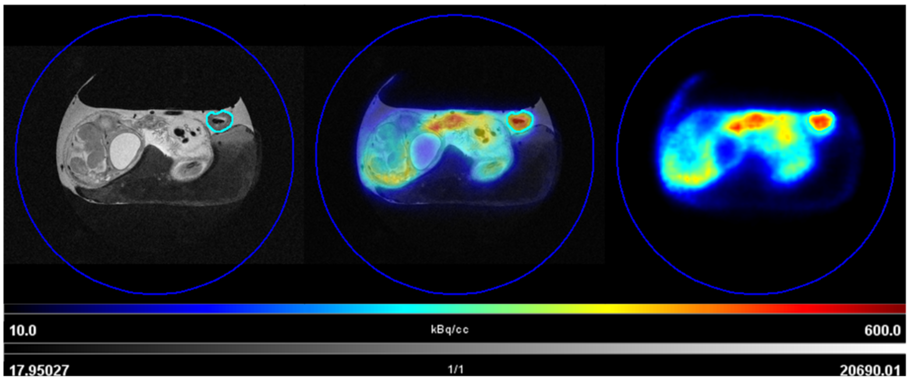

Figure S2. Example for VOI positioning for CAM-xenograft. Xenograft-VOIs were manually drawn based on the MR information from the T2-weighted TurboRARE scans. Left: T2-weighted TurboRARE image, right: PET, middle: fused PET and MR (axial planes are shown). Xenografts (turquoise VOI) are marked with a yellow arrow.

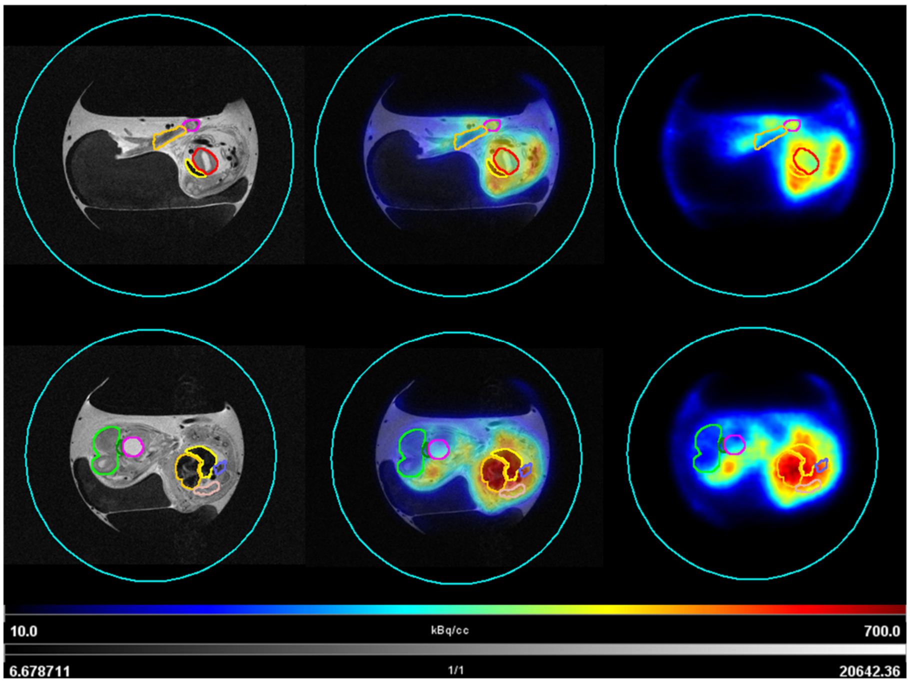

Figure S3. Example for VOI positioning for different organs and other tissues within the chicken egg. VOIs were manually drawn based on the MRI information from the T2-weighted TurboRARE scans. VOIs for joints and bones were manually drawn based on the fused PET/MR images. For albumen and yolk, a sphere, 2 mm in radius, was positioned in albumen or yolk, respectively. Total activity was calculated based on a spherical VOI with 30 mm radius around the whole egg. Left: T2-weighted TurboRARE images, right: PET, middle: fused PET and MR (axial planes are shown). Upper row: Bone (tarsometatarsus) – orange, joint (intertarsal) – pink, gizzard – red, liver – yellow, whole egg – turquoise. Lower row: Brain – green, eye – pink, heart – orange, liver – yellow, lung – rose, kidney – blue, whole egg – turquoise.

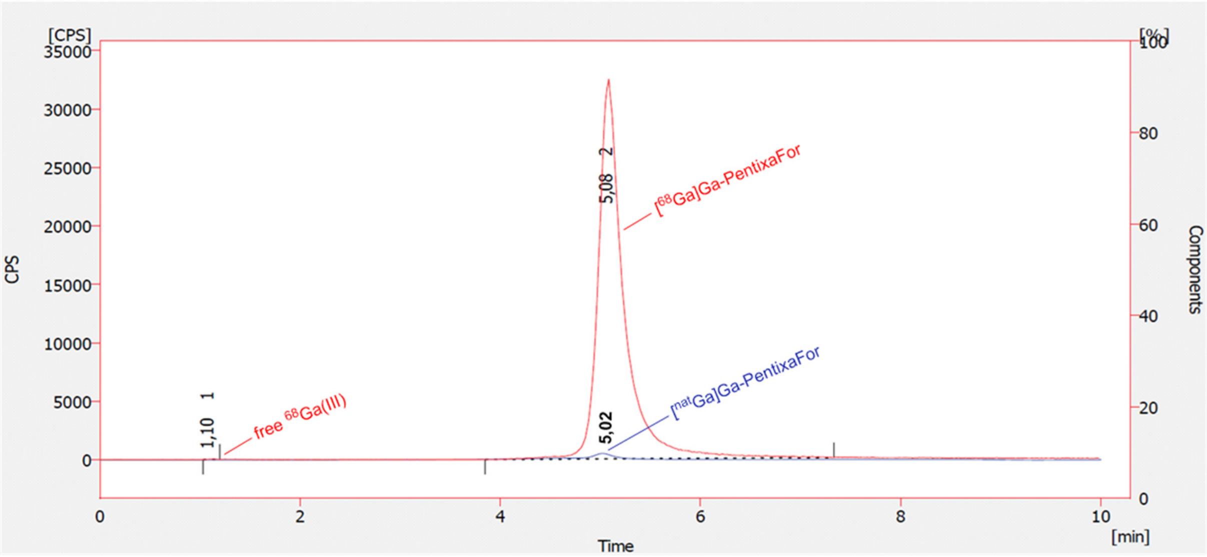

Result Table (Uncal - Detector 1)

	Reten. Time [min]	Area [CPS.s]	Height [CPS]	Area [%]	Height [%]	W05 [min]
1	1.100	182.000	39.400	0.0	0.1	0.08
2	5.083	580700.000	32477.933	100.0	99.9	0.25
	Total	580882.000	32517.333	100.0	100.0	

Result Table (Uncal - UV-220nm)

	Reten. Time [min]	Area [mAU.s]	Height [mAU]	Area [%]	Height [%]	W05 [min]
1	5.020	15010.114	523.184	100.0	100.0	0.24
	Total	15010.114	523.184	100.0	100.0	

Figure S4. Representative radio-HPLC chromatogram of [^{68}Ga]Ga-PentixaFor (red) with the co-injected standard [^{nat}Ga]Ga-Pentixafor (blue)

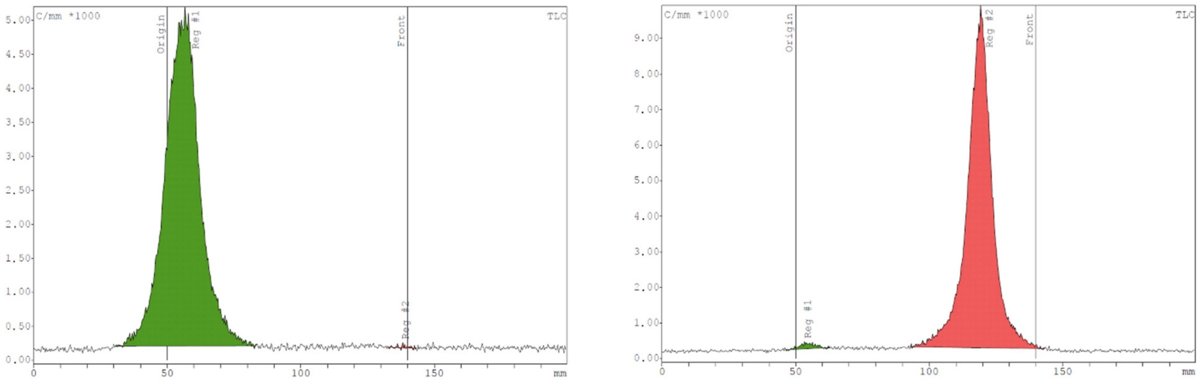

Integration TLC

Substance	R/F	%Total %	Type	Area Counts	%Area %
Reg #1	0.081	65.92	BB(	74202.57	99.89
Reg #2	0.985	0.07	BB(	80.64	0.11
Sum in ROI	-	-	-	74283.21	100.00
Total area	-	-	-	112572.00	-
Area (total) RF	-	-	-	79822.00	-

Integration TLC

Substance	R/F	%Total %	Type	Area Counts	%Area %
Reg #1	0.044	0.65	BB(	1002.4	0.98
Reg #2	0.770	65.85	BB(	100936.9	99.02
Sum in ROI	-	-	-	101939.3	100.00
Total area	-	-	-	153283.0	-
Area (total) RF	-	-	-	128714.0	-

Figure S5. Representative TLC-chromatogram of [^{68}Ga]Ga-Pentixafor using 0.1 M Na-citrate (left) and 1.0 M ammonium acetate/methanol (right) as solvent

Table S2. Uptake of [^{68}Ga]Ga-Pentixafor in HT29 and HCT116 cells.

Cellular fractions	HT29 cells [%AD/10^6 cells]	HCT116 cells [%AD/10^6 cells]
Total membrane binding	1.73 ± 0.44	0.20 ± 0.13
Unspecific membrane binding	0.08 ± 0.04	0.15 ± 0.08
Specific membrane binding	1.55 ± 0.39	0.07 ± 0.05
Total internalisation	0.46 ± 0.10	0.09 ± 0.04
Unspecific internalisation	0.07 ± 0.03	0.05 ± 0.03
Specific internalisation	0.40 ± 0.10	0.03 ± 0.02

Table S3. Uptake of [^{68}Ga]Ga-Pentixafor in CAM-xenografts derived from HT29 and HCT116 cells.

	HT29 CAM-xenografts [%TA/cc]	HCT116 CAM-xenografts [%TA/cc]
Total uptake	7.80 ± 1.88 (*n* = 7)	4.17 ± 1.29 (*n* = 9)
Unspecific uptake	4.05 ± 0.92 (*n* = 3)	3.34 ± 1.09 (*n* = 8)

Table S4. Accumulation of [^{68}Ga]Ga-Pentixafor in organs and other tissues within the chicken egg

VOI	Baseline condition [%TA/cc]	Blocking condition [%TA/cc]
Brain	4.73 ± 0.24	3.78 ± 0.18
Eyes	3.53 ± 0.22	3.10 ± 0.27
Liver	10.26 ± 1.08	10.49 ± 1.09
Heart	10.65 ± 1.03	10.00 ± 0.92
Kidney	10.18 ± 0.87	9.58 ± 0.52
Lungs	9.52 ± 0.66	8.83 ± 0.21
Gizzard	8.17 ± 0.88	7.53 ± 0.61
Joints	7.71 ± 0.23	7.27 ± 0.94
Bones	5.05 ± 0.91	5.76 ± 0.58
Albumen	1.35 ± 0.26	1.62 ± 0.57
Yolk	0.54 ± 0.56	0.38 ± 0.13

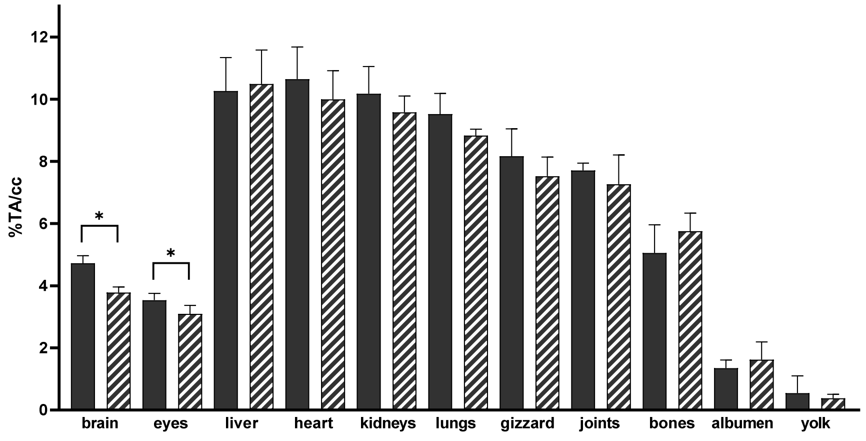

Figure S6. Distribution of [^{68}Ga]Ga-Pentixafor under baseline (filled bars) and blocking (bars with pattern) conditions. Data is depicted as % total activity per cm^3 (%TA/cc) and presented as mean ± SD (n = 4). Significant differences (p <0.05) are marked with an asterisk (*).

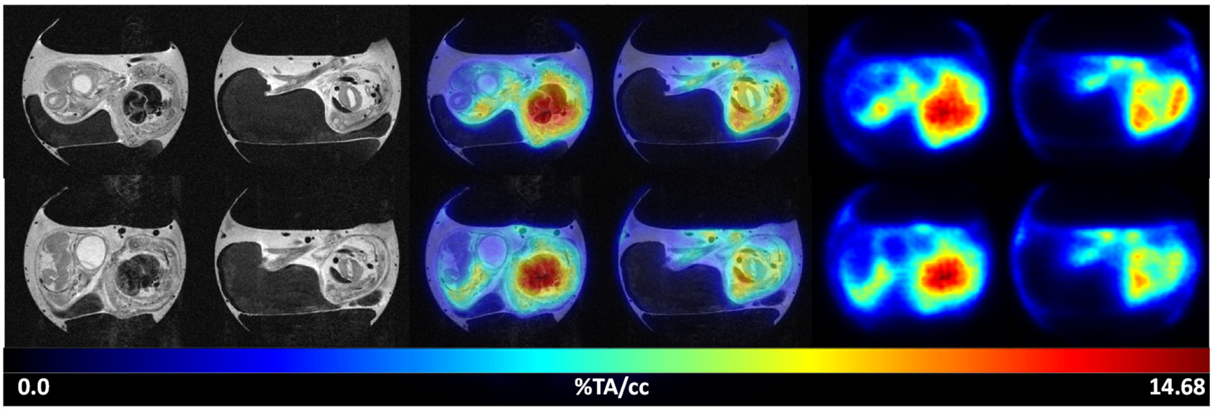

Figure S7. Representative images of [^{68}Ga]Ga-Pentixafor distribution in the chick embryonal organism. Upper row: [^{68}Ga]Ga-Pentixafor baseline after injection of 8.73 MBq (2.46 nmol peptide) into a CAM-vessel on EDD16. Lower row: [^{68}Ga]Ga-Pentixafor distribution under blocking conditions in the same egg. Imaging was performed on the following day (EDD17) after injection of 315 µg AMD3100 and 15.42 MBq [^{68}Ga]Ga-Pentixafor (1.75 nmol peptide). PET imaging was performed 60 min p.i. Left: T2-weighted TurboRARE image, right: PET, middle: fused PET and MR (axial planes are shown).

Table S5. Accumulation of 2-[^{18}F]FDG in embryonal organs as well as HT29 and HCT116 CAM-xenografts

VOI	Uptake [%TA/cc]
HT29-xenograft	9.12 ± 2.19
HCT116-xenograft	9.53 ± 1.22
Brain	7.27 ± 1.13
Eyes	6.31 ± 0.81
Liver	7.36 ± 1.18
Heart	6.86 ± 1.00
Kidneys	7.86 ± 0.39
Lungs	6.11 ± 1.00
Gizzard	5.63 ± 0.94
Joints	8.49 ± 1.53
Bones	5.40 ± 0.55
Albumen	1.56 ± 1.06
Yolk	0.36 ± 0.15

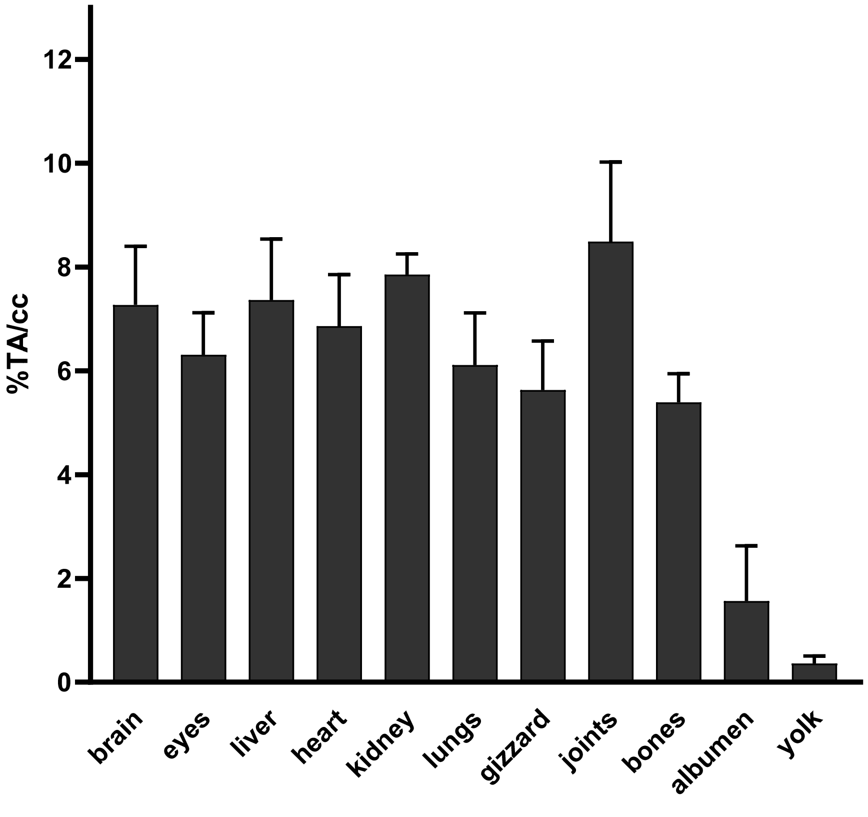

Figure S8. 2-[^{18}F]FDG distribution in chick embryonal organs, albumen and yolk. Data is depicted as %TA/cc and presented as mean ± SD (n = 3).

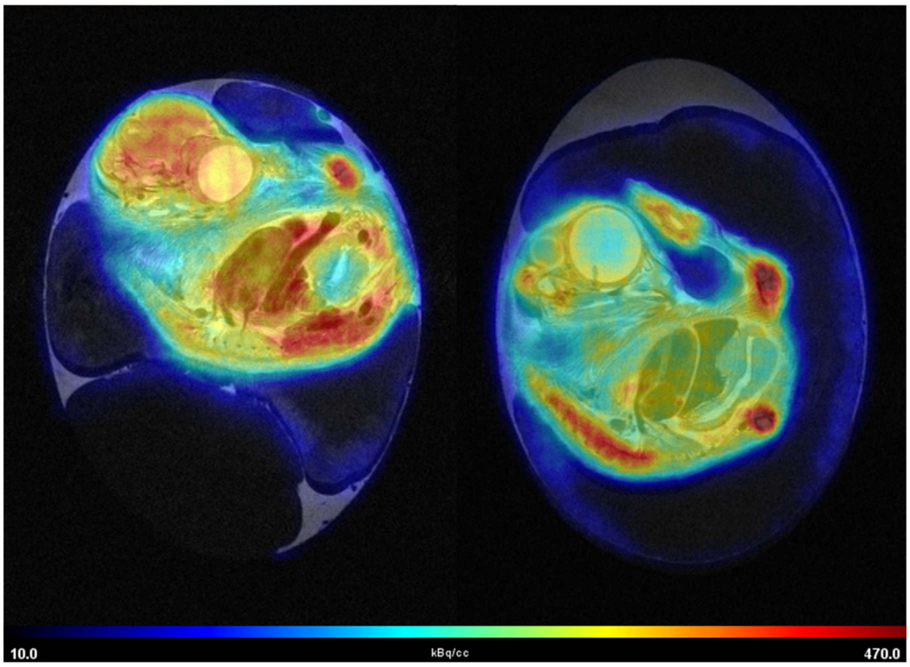

Figure S9. Selected PET/MR images of 2-[^{18}F]FDG uptake in two different subjects. Left: Imaging was performed on EDD18 60 min after injection of 8.94 MBq 2-[^{18}F]FDG using the radiopharmaceutical preparation 30 min after radiosynthesis. Right: Imaging was performed on EDD18 60 min after injection of 5.31 MBq 2-[^{18}F]FDG using the radiopharmaceutical preparation 6 h after radiosynthesis. In both images, a fused T2-weighted TurboRARE image and PET image in the coronal plane is shown.

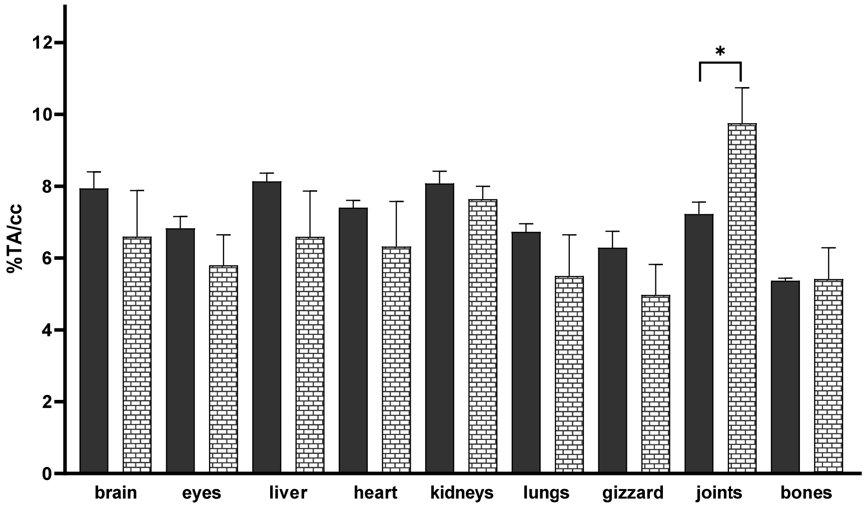

Figure S10. Comparison of 2-[^{18}F]FDG uptake into different organs and tissues of the chick embryo when performing the scan early (up to 2.5 h after radiolabelling, filled bars) or later (more than 5.5 h after radiolabelling, bars with pattern) during the day. Data is depicted as %TA/cc and presented as mean ± SD ($n \geq 3$). Significant differences ($p < 0.05$) are marked with an asterisk (*).
